# Dispositional mindfulness and its relationship to exercise motivation and experience

**DOI:** 10.3389/fspor.2022.934657

**Published:** 2022-11-29

**Authors:** Sarah Lynn, Medha Kumari Satyal, Alana J. Smith, Noor Tasnim, Daphne Gyamfi, Daniel F. English, Wendy A. Suzuki, Julia C. Basso

**Affiliations:** ^1^Department of Human Nutrition, Foods, and Exercise, Virginia Tech, Blacksburg, VA, United States; ^2^Translational Biology, Medicine, and Health Graduate Program, Virginia Tech, Blacksburg, VA, United States; ^3^School of Neuroscience, Virginia Tech, Blacksburg, VA, United States; ^4^Center for Neural Science, New York University, New York, NY, United States; ^5^Center for Health Behaviors Research, Fralin Biomedical Research Institute at VTC, Roanoke, VA, United States

**Keywords:** mindfulness, meditation, self-regulation, exercise, exercise dependence, mindfulness-based stress reduction

## Abstract

Mindfulness is the psychological state of staying attuned to the present moment, without ruminating on past or future events, and allowing thoughts, feelings, or sensations to arise without judgment or attachment. Previous work has shown that heightened dispositional mindfulness is associated with the awareness of the importance of exercise, exercise self-efficacy, exercise motivation, and self-reported exercise level. However, more methodologically rigorous studies are needed to understand the relationship between mindfulness and the psychological mechanisms related to exercise motivation, including the identification of why individuals are motivated to engage in exercise, the subjective experience of exercise, and the propensity for exercise dependence and addiction. In this cross-sectional investigation, we utilized the framework of the Self-Determination Theory to examine the hypothesis that heightened dispositional mindfulness (as measured by the Mindful Attention Awareness Scale) would be associated with increased levels of exercise motivation that were derived by higher levels of autonomous self-regulation. Individuals were recruited from urban areas who self-reported either low (exercising 2 or fewer times per week for 20 min or less; *n* = 78) or moderate (exercising 1 or 2 times per week for 20 min or more; *n* = 127) levels of exercise engagement. As hypothesized, heightened dispositional mindfulness was significantly associated with heightened levels of exercise self-determination as measured by the Behavioral Regulations in Exercise Questionnaire, with this effect being driven by negative associations with amotivation, external regulation, and introjected regulation. Additionally, we found that heightened dispositional mindfulness was associated with lower levels of psychological distress upon exercise and decreased exercise dependence/addiction. Overall, increased dispositional mindfulness may support a healthy relationship with exercise. These findings have implications for the utility of mindfulness interventions to support the regulation of exercise behaviors in service of enhancing exercise motivation and engagement.

## Introduction

Physical activity is defined as any bodily movement produced by skeletal muscles that results in energy expenditure over basal levels (1). Engagement in physical activity is imperative for physical and psychological health, as opposed to sedentary behavior that leads to a range of clinical issues including obesity, diabetes, cancer, mood disorders, and shortened life span (2–5). The American Heart Association and Center for Disease Control recommend engaging in at least 150 min of moderate-intensity or 75 min of vigorous-intensity aerobic exercise per week to reduce risk of chronic disease and increase physical and mental quality of life (1). However, only 23% of Americans obtain this level of physical activity (6–8). Despite rampant public health campaigns across America (e.g., Michelle Obama's Let's Move initiative) and general knowledge of the benefits of physical activity, engagement in physical activity remains an issue (9–11). Additionally, longitudinal studies seeking to increase physical activity levels show limited success, with high dropout rates and low levels of adherence (11–13). These challenges are particularly relevant for clinical populations with low levels of physical activity and high levels of sedentary behavior such as those with obesity and type 2 diabetes, where exercise may be perceived as difficult, uncomfortable, or unrewarding (14–18).

Therefore, understanding the psychological mechanisms underlying physical activity is necessary to understand ways to support this important health behavior. We have previously shown that obtaining a habitual physical activity regimen in a rodent model requires long-term engagement (i.e., at least 3 weeks) in physical activity, with this behavior being supported by reward-related brain circuits including the nucleus accumbens and prefrontal cortex (19, 20). In humans, psychological factors such as self-efficacy, self-regulation (e.g., delay discounting), and the affective response to exercise may contribute to engagement in physical activity, but more research is needed in this area (17, 21–23). Mindfulness is a psychological concept that has received much attention in recent years due to its involvement in promoting health-behavior change (24–27). A growing body of evidence suggests that mindfulness can affect an individual's self-regulation, which includes attention, cognitive control, emotion regulation, self-related processes, motivation, and learning (27).

Mindfulness is the psychological state of staying attuned to the present moment, without ruminating on past or future events, and allowing thoughts, feelings, or sensations to arise without judgment or attachment. Mindfulness-based interventions such as mindfulness-based stress reduction have been utilized to improve health behaviors such as smoking, drinking, and overeating, with various degrees of success (28–30). In regard to exercise motivation, previous work has shown that heightened levels of dispositional mindfulness are associated with the awareness of the importance of exercise, exercise self-efficacy, exercise motivation, and self-reported exercise level (31–33). However, more research is needed to understand the relationship between mindfulness and the psychological mechanisms related to exercise motivation, including the identification of why individuals are motivated to engage in exercise, the subjective experience of exercise, and the propensity for exercise dependence and addiction.

Self-Determination Theory (SDT) argues that autonomous and controlled motivation drive human behavior (34–36). This theory is unique because it emphasizes the importance of type, rather than amount, of motivation, in human behavioral outcomes. SDT assumes that all humans are naturally self-motivated; however, their social environments can either amplify or limit that sense of motivation because of their inherent need to “feel competent, autonomous, and related to others” (37). SDT has been investigated in a wide range of environments, including schools, workplaces, and clinics, and several validated tools have been developed to assess motivation through the lens of SDT.

Therefore, in this cross-sectional study we examined the relationship between mindfulness and various aspects of exercise motivation and psychology based on the perspective of SDT. We gathered data from a cohort of healthy adults with varying levels of exercise motivation from urban populations. We hypothesized that heightened dispositional mindfulness would be associated with increased levels of exercise motivation that are driven by more autonomous self-regulation to exercise and a more positive psychological experience of exercise. Findings are reported in the context of future clinical interventions that may be utilized to support exercise motivation in both healthy and clinical populations.

## Methods

### Participants

A total of *n* = 205 participants were recruited from Austin, TX, and New York, NY through flyers and online advertisements. All participants were healthy males and females between the ages of 25 and 59, with English as their primary language (Table 1). Participants were excluded if they currently smoked, had back, hip, or knee issues, or other preexisting health conditions that made exercise difficult or unsafe. Participants were also excluded if they were diagnosed with and/or took medication for psychiatric or neurological conditions, including anxiety, depression, bipolar disorder, schizophrenia, substance use disorders, eating disorders, or epilepsy. Before participation, all participants gave their informed consent. All study documentation and data collection methods were approved by and in compliance with the New York University Committee on Activities Involving Human Subjects.

**Table 1:** Participant demographics: Age presented as Mean (SEM).

***N***	**205**
Age	35.8 (0.67)
Sex	
% Female	74.1
% Male	25.9
Education	
% High school	3.9
% Some college or 2-year degree	18.5
% Bachelor's degree	47.8
% Advanced or professional degree	28.3
% No response	1.5

N = 204 for age due to 1 missing value. All other variables are presented as percentages.

### Procedures

Participants in this study self-reported either low (*n* = 78) or moderate (*n* = 127) levels of exercise. Low levels of exercise were defined as exercising for 20 min or less, 2 or fewer times per week. Moderate levels of exercise were defined as exercising more than 20 min once or twice a week. Participants were asked to complete a series of self-report questionnaires regarding mindfulness and exercise motivation.

### Self-reported assessments

Participants were instructed to complete all self-report assessments (described below) at home while refraining from alcohol or illicit substances. If participants did not complete the assessment before coming into the laboratory, they were instructed to complete the assessment in the laboratory.

### The mindful attention awareness scale (MAAS)

The Mindful Attention Awareness Scale (MAAS) measures dispositional mindfulness (attention to and awareness of present experiences and events) (38). This scale consists of 15 items scored on a 6-point Likert scale. Participants respond with how often they experience certain events on a scale from “almost always” to “almost never.” All questions are summed for a total score. Higher scores indicate heightened levels of dispositional mindfulness. In a general adult sample, Cronbach's alpha is 0.87 indicating strong internal consistency.

### Behavioral regulation in exercise questionnaire (BREQ-2)

The BREQ-2 is a 19-item scale used to measure participants' motivation to engage in exercise (39). Scale items are scored on a 5-point Likert scale. Scoring requires a mean of each subscale (i.e., external regulation, introjected regulation, identified regulation, intrinsic regulation, and amotivation). Cronbach's alpha for each subscale is as follows: external regulation (α = 0.76), introjected regulation (α = 0.81), identified regulation (α = 0.84), intrinsic regulation (α = 0.94), and amotivation α = 0.85) (40). The Relative Autonomy Index (RAI) indicates the degree to which participants are self-determined to exercise (41). This scale, with a range of −24 to 20, is calculated using the following formula based on BREQ subscales: [amotivation ^*^ (−3)] + [external regulation ^*^ (−2)] + [introjected regulation ^*^ (−1)] + (identified regulation ^*^ 2) + (intrinsic regulation ^*^ 3). Lower scores indicate less autonomous motivation, while higher scores indicate higher autonomous motivation.

### Subjective exercise experience scale (SEES)

SEES measures the psychological response to exercise, assessing positive well-being, psychological distress, and fatigue (42). This scale consists of 12 items. Participants rated how strongly they experienced each feeling on a 7-point Likert scale ranging from “not at all” to “very much so.” Internal consistency is high, as measured by Cronbach's alpha, for each of the 3 factors: positive well-being (α = 0.86), psychological distress (α = 0.85), and fatigue (α = 0.88). Individual factor scores are summed. Higher scores equate to higher levels of positive well-being, psychological distress, or fatigue.

### Exercise motives inventory (EMI-2)

The Exercise Motives Inventory examines factors that influence or motivate exercise participation (43). This scale consists of 51 items scored on a 6-point Likert scale measuring fourteen subscales (stress management, revitalization, enjoyment, challenge, social recognition, affiliation, competition, health pressures, ill-health avoidance, positive health, weight management, appearance, strength & endurance, and nimbleness). Scores for each subscale are calculated by taking the mean of appropriate items. Cronbach's alpha for this scale ranges from α = 0.686 to α = 0.954 for each subscale.

### The exercise causality orientations scale (ECOS)

The Exercise Causality Orientations Scale examines how different individuals seek to be controlled or autonomous in their behavioral regulation regarding exercise (44). ECOS recognizes three types of individual orientations: autonomy orientation (α = 0.73), control orientation (α = 0.77), and impersonal orientation (α = 0.71). Confirmatory factor analysis identified a 7-scenario model (chi square = 445; df = 165; CFI = 0.96; SRMR = 0.06; RMSEA = 0.05). Autonomy-oriented individuals regulate their behavior based on personal goals and beliefs and look for opportunities to be self-determined. Control-oriented individuals rely on extrinsic motivations such as deadlines or rewards to regulate their behavior. Finally, impersonal-oriented individuals believe that they cannot regulate their behavior to produce a specific outcome since they believe behavioral outcomes are out of their control. This scale contains seven written scenarios followed by three responses. Participants respond to each scenario on a 7-point Likert scale.

### Locus of causality for exercise scale (LCES)

The LCES examines the extent to which people feel that they are forced to exercise rather than freely choosing to do so (45). Feeling forced to exercise includes feeling external or self-imposed pressures. Individuals with this mindset have an external perceived locus of causality. When individuals feel they are engaging in a behavior of their own volition, they have an internal perceived locus of causality. This scale consists of 3 items scored on a 7-point Likert scale, which are averaged for the total LCES score. Higher scores indicate greater self-determination (more internal perceived locus of control). Cronbach's alpha measuring internal consistency for this scale is α = 0.83.

### The exercise addiction inventory (EAI)

The Exercise Addiction Inventory screens for exercise addiction (46). This scale consists of six items on a 5-point Likert scale ranging from “strongly disagree” to “strongly agree.” Each item corresponds to six addiction components: salience, conflict, mood modification, tolerance, withdrawal, and relapse. A higher score indicates a higher degree of exercise addiction. The internal reliability of this scale is good, with Cronbach's alpha = 0.84.

### The exercise dependence scale (EDS)

The EDS uses criteria from the DSM-IV diagnosis of substance dependence to assess exercise dependence. The scale consists of 21 items regarding feelings about exercising. Participants respond on a 6-point Likert scale regarding their frequency of experiencing thoughts/feelings ranging from “never” to “always.” The scale is broken down into seven components: withdrawal effects, continuance, tolerance, lack of control, reduction in other activities, time, and intention effects. Cronbach's alpha for this scale is 0.909 (47).

### Statistical analysis

Relationships between mindfulness and exercise motivation were probed using Pearson's product-moment correlations. Bonferroni corrections were completed for each family of statistical tests, and statistical significance was determined for each family of statistical tests based on that value. We consider trends as any statistically significant relationship that fell in between <0.05 and the Bonferroni corrected *p*-value. Mean age and demographic frequencies were calculated to display this study population's characteristics. An alpha value of 0.05 was utilized to determine statistical significance. IBM SPSS Statistics Version 27.0 was utilized for all statistical analyses (48).

## Results

### Demographics

Data analysis included *n* = 205 participants. Our participants were on average middle-aged (35.8 ± 0.67 years), primarily female, and highly educated (Bachelor's degree or higher).

### Behavioral regulations in exercise questionnaire (BREQ-2)

**Figure 1:**
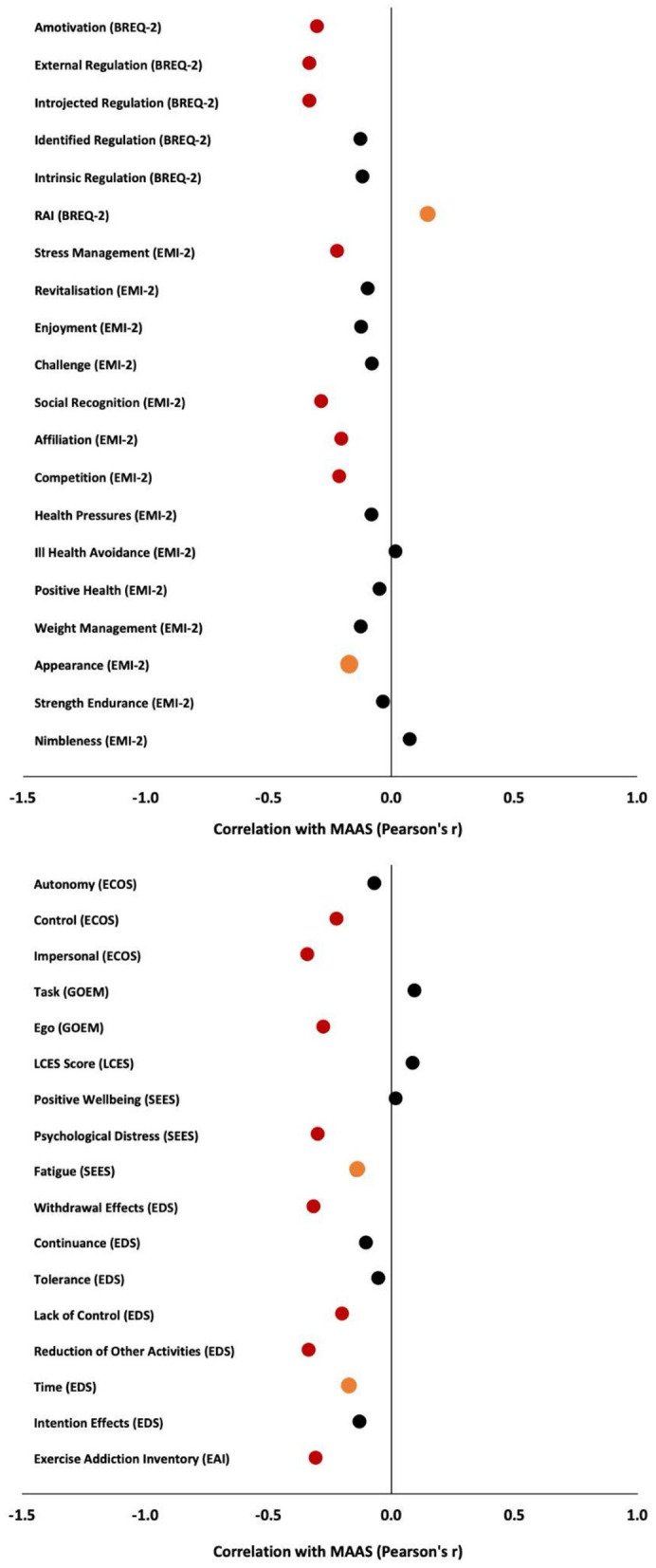
Correlations between Mindful Attention Awareness Scale (MAAS) and study measures of interest. Red circles indicate significance, orange circles indicate trends toward significance, and black circles indicate non-significance.

Dispositional mindfulness was negatively and significantly correlated with several factors of the BREQ-2, including amotivation (r = −0.302, <0.001), introjected regulation (r = −0.333, <0.001) and external regulation (r = −0.333, <0.001). Additionally, MAAS showed a positive and significant trend with the relative autonomy index (RAI) (r = 0.150, *p* = 0.032) (Figure 1). No significant correlations were found for any other BREQ-2 subscales, including identified regulation and intrinsic regulation (*p* > 0.008).

### Exercise motivations inventory (EMI-2)

Dispositional mindfulness as assessed by the MAAS was negatively and significantly correlated with several factors of the EMI including stress management (r = −0.220, *p* = 0.002), social recognition (r = −0.285, <.001), affiliation (r = −0.204, *p* = 0.003), and competition (r = −0.210, *p* = 0.003), with appearance showing a trend toward significance (r = −0.177, *p* = 0.011) (Figure 1). No significant correlations were found for other EMI subscales, including enjoyment, revitalization, challenge, health pressures, ill health avoidance, positive weight management, strength enjoyment, or nimbleness (*p* > 0.004).

### Exercise causality orientation scale (ECOS)

Dispositional mindfulness was negatively and significantly correlated with several factors of the ECOS, including control (r = −0.223, *p* = 0.001) and impersonal (r = −0.340, <0.001) (Figure 1). No significant correlation was found for autonomy (*p* > 0.017).

### Goal orientations and exercise motivation scale (GOEM)

Dispositional mindfulness was negatively and significantly correlated with the ego subscale (r = −0.277, <0.001) but not the task subscale (*p* > 0.025) (Figure 1).

### Locus of causality for exercise scale (LCES)

Dispositional mindfulness was not associated with the LCES score (*p* > 0.05) (Figure 1).

### Subjective exercise experiences scale (SEES)

Dispositional mindfulness was negatively and significantly correlated with psychological distress (r = −0.298, <0.001), with a trend showing for fatigue (r = −0.140, *p* = 0.046), and no significant effect for positive well-being (*p* > 0.017) (Figure 1).

### Exercise dependence scale (EDS)

Dispositional mindfulness was negatively and significantly correlated with several factors of the EDS, including average EDS score (r = −0.278, <0.001), withdrawal effects (r = −0.316, <0.001), lack of control (r = −0.200, *p* = 0.004), and reduction of other activities (r = −0.334, <0.001) (Figure 1), with a trend showing for time (r = −0.170, *p* = 0.015). No significant correlations were found for other EDS subscales, including continuance, tolerance, and intention effect (*p* > 0.006).

### Exercise addiction inventory (EAI)

Dispositional mindfulness was negatively and significantly correlated with EAI total (r = −0.306, <0.001) (Figure 1).

## Discussion

In this cross-sectional investigation, we examined the relationship between dispositional mindfulness and various psychological factors related to exercise motivation including the reasons for exercise engagement, the subjective experience of exercise, and exercise dependence and addiction. Supporting our hypothesis, heightened dispositional mindfulness was associated with increased exercise motivation. Interestingly, high levels of mindfulness were associated with lower levels of controlled motivation to exercise, psychological distress upon exercise, and exercise dependence/addiction. Overall, our results indicate that increased dispositional mindfulness may support a healthy relationship with exercise.

### Dispositional mindfulness and autonomous vs. controlled regulation for exercise

The current study found that individuals with greater dispositional mindfulness feel more self-determined to exercise. Specifically, greater dispositional mindfulness was associated with less amotivation, introjected regulation, and external regulation for exercise. More specifically, individuals with high levels of dispositional mindfulness are less likely to engage with exercise because of motivations that are driven by either 1) an internal sense of compulsion, pressure, or guilt (i.e., introjected regulation), or 2) external contingencies such as punishments or rewards (i.e., external regulation) (49). Additionally, the results show that individuals with greater dispositional mindfulness are less motivated by external goals to exercise, including ego-orientation or the comparison of self to others (ego subscale of GOEM), as well as control orientation or the imposition of either internal or external events to control one's behavior (control orientation scale of ECOS). Furthermore, individuals with heightened mindfulness are less likely to feel unable to regulate their exercise behavior (impersonal orientation scale of ECOS). This is the first study to examine the relationship between dispositional mindfulness and internal/external motivators of exercise through the simultaneous administration of multiple self-reported assessments related to exercise motivation from the perspective of self-determination theory.

We speculate that our findings may be due to the fact that the psychological state of mindfulness is associated with enhanced self-regulation, which can include modulation of emotional states as well as acceptance of these internal states (50–52). Subsequently, mindfulness is associated with lower engagement in unhealthy behaviors due to such negative internal states (27, 53). This means that in mindful individuals, negative internal states or external pressures will have less influence over the behavioral outcome, which in this case is physical activity behavior. This is evidenced in our data showing that high levels of dispositional mindfulness are related to low levels of amotivation to exercise, which is a state characterized by a lack of intention or resistance to engage in a behavior (49).

Our findings are similar to those reported in the literature, but highlight the relationship between dispositional mindfulness and exercise motivation through a different lens. Other studies have shown a positive association between dispositional mindfulness and intrinsic motivators of exercise (54–56). Although our study did not observe this positive relationship, there was a negative association between mindfulness and either negative internal drivers or external motivators. Both of these relationships have similar clinical implications. An understanding of the relationship between dispositional mindfulness and heightened exercise motivation, either through increased intrinsic motivation or reduced extrinsic motivation, can guide the design of future mindfulness interventions aimed at increasing physical activity levels. For instance, mindfulness interventions focused on an individual's desires or needs (e.g., self-compassion based meditation) rather than an external reward (e.g., monetary incentives) could promote fidelity toward increased levels of physical activity.

### Dispositional mindfulness and reasons for exercise engagement

To develop a better understanding of how dispositional mindfulness is related to one's reasons for exercising, we utilized the EMI-2 (43). Heightened dispositional mindfulness was associated with lower levels of exercise engagement due to extrinsic factors such as social recognition, affiliation, and competition. Additionally, heightened dispositional mindfulness was negatively associated with both appearance (trend) and stress management, harking back to our observations regarding mindfulness being negatively associated with negative internal states that drive exercise. The negative correlation between these factors and dispositional mindfulness supports our hypothesis based on SDT, which states that human actions are driven by the type of motivation, specifically autonomous or controlled, rather than the amount of motivation (35). Specifically, those with high levels of mindfulness report lower levels of both negative internally-driven and externally-driven reasons for exercise engagement. That is, more mindful individuals demonstrate lower levels of controlled regulation to exercise.

As social recognition, affiliation, and competition subscales all involve the influence of at least one other person, our results suggest that mindfulness may play a role in diminishing the negative or judgemental influence of others (57, 58), limiting this external influence on the motivation to exercise. This is in line with other related work showing that dispositional mindfulness is predictive of exercise motivation, with this effect being fully mediated by lowered negative affect and shame in response to health messages to encourage physical activity and discourage sedentary behavior (31). Other researchers suggest that mindfulness skills may promote intrinsic motivation for physical activity and that mindfulness and intrinsic motivation independently promote exercise self-efficacy, which is a key element in the self-determination to exercise (33). The relationships among mindfulness and both intrinsic motivation and extrinsic motivation to exercise are clearly inter-related suggesting future research should include mediation/moderation analysis to clarify these relationships.

### Dispositional mindfulness and the subjective response to exercise

We also found that heightened dispositional mindfulness was negatively associated with feelings of psychological distress (e.g., crummy, awful, miserable, discouraged) in response to exercise. This is the first time that the SEES has been examined in relation to mindfulness. This finding has important clinical implications and suggests that cultivating mindfulness through mindfulness-based interventions may reduce perceived psychological barriers to exercise, though future randomized control trials are needed to determine a causal effect. This finding may be especially important for clinical populations such as those with obesity and diabetes who may experience psychological distress in response to exercise (59, 60).

Our findings support our initial hypothesis and are in line with previous research. For example, a recent study found that greater dispositional mindfulness reduced the perceived barriers to exercise by reducing psychological distress in help-seeking young adults (61), while another found that satisfaction with exercise mediated the effect of mindfulness on engagement in physical activity (62). Additionally, an acute interventional study found that compared to a control walking condition, mindful walking was associated with higher levels of positive affective valence, enjoyment and mindfulness of the body, and attentional focus (63). Longitudinal studies have also shown that participants engaging in a mindfulness intervention maintain more positive affective responses to exercise and continue to engage in more days of exercise at follow up compared to controls (64). Additionally, the affective response to exercise predicts engagement in physical activity and this relationship is mediated by the intrinsic motivation for exercise (65). In line with this body of research, we observed that increasing mindfulness may help support the affective response to exercise and thus encourage engagement with physical activity behaviors. Future studies are needed to examine the relationships between the subjective exercise experience and mindfulness following both acute and chronic bouts of exercise.

### Dispositional mindfulness and exercise dependence and addiction

Heightened levels of dispositional mindfulness were associated with lower levels of exercise dependence and addiction, specifically in the areas of lack of control, withdrawal, and reduction of other activities. These findings indicate that a more mindful individual is less likely to experience symptoms or characteristics of exercise dependence and/or addiction. This is the first time that mindfulness has been studied in relation to exercise dependence/addiction. As mindfulness-based interventions are commonly used for individuals with substance use disorders (66, 67), this work suggests that mindful interventions may also be helpful for individuals with exercise addictions, such as individuals with eating disorders (e.g., anorexia nervosa).

Recent work has found that exercise addiction may be an integral component of orthorexia nervosa, which is an unspecified feeding and eating disorder that is characterized by the fifth edition of the Diagnostic and Statistical Manual of Mental Disorders by obsessive and compulsive behaviors related to healthy eating (68). Individuals with orthorexia symptomatology have heightened levels of exercise motivation, which are driven by factors associated with improving physical and mental health (69). However, these behaviors may lead to exercise addiction, which is characterized by a compulsive need to engage in exercise despite injury, illness, or other ailments. Our research indicates that enhancing mindfulness may lead to a healthier relationship with exercise, perhaps driven by the fact that mindfulness is associated with heightened interoception and self-awareness (52, 70–72). This heightened level of mindfulness may lead an individual to listen to their own physical and mental needs, thus developing a healthy relationship with health-related behaviors such as eating, drinking, and exercising.

### Limitations and future directions

While this study provides new insights into the relationships between mindfulness and exercise motivations and attitudes, several limitations are worth noting. First, the study is cross-sectional, limiting the ability to assess causal relationships and longitudinal changes. Additionally, data collection occurred in two urban areas, New York, New York, and Austin, Texas, and thus may not generalize to the broader population. Additionally, limited demographic factors were collected (i.e., age and education). The majority of our study participants were female (74.1%) and a significant percentage were college educated (76.1%); future studies should consider collecting data on more diverse populations. All data used in this study were self-reported, which has inherent limitations. Self-report data often show acquiescence and social desirability biases and are subject to participant fatigue.

Despite these limitations, our data indicate that mindfulness is associated with heightened self-autonomy for exercise motivation and an overall healthier relationship with exercise. Future work is warranted to test the utility of mindfulness interventions, such as mindful meditation, to promote engagement in exercise. Moreover, it is worth examining how mindfulness affects motivation for specific types of exercises based on an individual's preferences. Additionally, as the neural mechanisms of mindfulness and exercise motivation share some overlapping features (e.g., reward circuitry structures such as the prefrontal cortex) (19, 52), future functional magnetic resonance imaging studies are needed to identify the neural correlates of how mindfulness may drive exercise motivation.

## Conclusions

In this cross-sectional study based on the framework of SDT, heightened dispositional mindfulness was related to an increased self-determination for exercise, which was driven by lower levels of introjected and external regulation as well as lower amotivation to exercise. Further, mindfulness was associated with an improved psychological response to exercise and a lower likelihood of reporting an exercise dependence or addiction. Hence, it is possible that increased mindfulness could enhance the positive affect associated with exercise and drive motivation. Overall, our findings suggest that increased mindfulness is associated with an improved drive to exercise and a healthier relationship with exercise. Future research is needed to examine whether increasing mindfulness through mindfulness-based practices such as yoga or meditation will help to increase the motivation for exercise. This work has implications for clinical populations that find exercise difficult or uncomfortable, as it suggests that increasing mindfulness may decrease barriers to engage in physical activity.

## Data availability statement

The raw data supporting the conclusions of this article will be made available by the authors, without undue reservation.

## Ethics statement

The studies involving human participants were reviewed and approved by New York University Committee on Activities Involving Human Subjects. The patients/participants provided their written informed consent to participate in this study.

## Author contributions

WS, JB, and DE contributed to the conceptualization and design of the study. JB collected all study data. JB, SL, MS, AS, NT, and DG all contributed to data analysis, data interpretation, and writing of the manuscript. All authors reviewed and approved the final version of the manuscript.

## Funding

JB is an iTHRIV Scholar. The iTHRIV Scholars Program was supported in part by the National Center for Advancing Translational Sciences of the National Institutes of Health under Award Numbers UL1TR003015 and KL2TR003016.

## Conflict of interest

The authors declare that the research was conducted in the absence of any commercial or financial relationships that could be construed as a potential conflict of interest.

## Publisher's note

All claims expressed in this article are solely those of the authors and do not necessarily represent those of their affiliated organizations, or those of the publisher, the editors and the reviewers. Any product that may be evaluated in this article, or claim that may be made by its manufacturer, is not guaranteed or endorsed by the publisher.
